# A novel interpretable machine learning algorithm to identify optimal parameter space for cancer growth

**DOI:** 10.3389/fdata.2022.941451

**Published:** 2022-09-12

**Authors:** Helena Coggan, Helena Andres Terre, Pietro Liò

**Affiliations:** ^1^Department of Mathematics, University College London, London, United Kingdom; ^2^Department of Computer Science and Technology, University of Cambridge, Cambridge, United Kingdom

**Keywords:** cancer, neural networks, white-box machine learning, interpretability, parameter optimization

## Abstract

Recent years have seen an increase in the application of machine learning to the analysis of physical and biological systems, including cancer progression. A fundamental downside to these tools is that their complexity and nonlinearity makes it almost impossible to establish a deterministic, *a priori* relationship between their input and output, and thus their predictions are not wholly accountable. We begin with a series of proofs establishing that this holds even for the simplest possible model of a neural network; the effects of specific loss functions are explored more fully in Appendices. We return to first principles and consider how to construct a physics-inspired model of tumor growth without resorting to stochastic gradient descent or artificial nonlinearities. We derive an algorithm which explores the space of possible parameters in a model of tumor growth and identifies candidate equations much faster than a simulated annealing approach. We test this algorithm on synthetic tumor-growth trajectories and show that it can efficiently and reliably narrow down the area of parameter space where the correct values are located. This approach has the potential to greatly improve the speed and reliability with which patient-specific models of cancer growth can be identified in a clinical setting.

## 1. Introduction

The application of neural networks to the modeling of cancer has seen a flood of interest in recent years (Sanoob et al., 2016; Hsu et al., 2018; Ghazani et al., 2021; Kwak et al., 2021; Kumar et al., 2022). The hope is to be able to use patient-specific data to generate accurate predictions of tumor growth and treatment response, in order to guide the clinician in their prognosis and choice of treatment regime (Rockne et al., 2019; Kumar et al., 2022). From a modeling perspective, a tumor is a system of interacting objects (tumor cells, fibroblasts, etc.) which influence each other's behavior according to certain rules. It should therefore be possible to use tumor-growth data to derive a system of equations to describe the trajectory of cancer, which can then be extrapolated into the future to predict the course of a particular disease. Over the last few years, neural networks have become the natural first choice of most scientists when tasked with extracting such equations from large datasets (Benzekry, 2020; Kurz et al., 2021). However, when we resort to machine learning to build models and predict the behavior of any system, we sacrifice a crucial attribute: *explainability*. The sheer vastness of a neural network, which may contain many tens of thousands of continually-adjusted interacting weights, makes the effort of deducing the impact of any single component on a network's output almost impossible. In addition, we must consider the neural network's various nonlinearities, which interfere with any attempt to construct an analytically solvable description of its processes (and thus to account for its decision-making). One example is the common Rectified Linear Unit (ReLU), and its many cousins [the parameterized ReLU (Xu et al., 2015), the “leaky” ReLU (Maas et al., 2013), etc.], which may or may not act on an input as it makes its way through the system. Any attempt to construct a gradient of the output with respect to the input will have to contend with the resulting discontinuities. Less analytically troublesome, but still exhausting, are backpropagation algorithms: ADAM (Kingma and Ba, 2014), for instance, adjusts each weight not simply in response to its current effect on the output but to all of its past effects, which will create a new set of complex nonlinearities in any differential equation aimed at describing a the workings of a network.

The best that can be hoped for, then, is to gain a “general idea” of the effect of each network attribute, using hyperparameter tuning (Yuan et al., 2021). This is an obviously risky approach: sampling a few points in the hyperspace of all possible hyperparameter values does not give us a complete picture of the dependence of the output on our choice of values. Without a complete picture of this dependence, we can never be sure that the relationships predicted by a network reflect physical reality or are simply a product of its own internal calibration. This is the crucial issue, and why, as long as a neural network remains a “black box,” its output can never be fully understood or trusted, especially in a clinical setting where the results of a model may guide cancer treatment and thus affect a patient's length and quality of life. A lack of explainability is a significant impediment to the adoption of machine learning and other computational approaches in a clinical setting. It also hinders the clinician's ability to fully interact with and analyse ML-derived predictions: not knowing where they come from, it is very difficult to rigorously deduce what any set of values assigned to a tumor “mean,” or to “sanity-check” them against clinical expertize. To reliably incorporate computational methods into cancer treatment, we must either develop some picture of the workings of a neural network, or move away from stochastic gradient descent altogether, to an algorithmic approach whose decision-making processes are transparent and accountable. A great deal of interesting work has been done in recent years to achieve this first goal, attempting to render explainable the workings of black-box neural networks (Rudin, 2019; Kazhdan et al., 2020; Dujon et al., 2021; Magister et al., 2021). The general approach of such papers is either to deduce the emergent rules of the neural network from its behavior, or to induce such strong biases in its workings that it is naturally directs to the correct area of parameter hyperspace (as with the physics-inspired neural networks discussed in Karniadakis et al., 2021). Such *a posteriori* attempts to harness or constrain the chaotic nonlinear workings of a neural network, however, are no replacement for an *a priori* understanding of its rules and aims. Without this, no result derived from such a network can be considered mathematically rigorous, which becomes an increasingly serious problem as the area of application approaches the hard sciences. The aim of this paper is to explore the difficulties inherent in this promising research, and to place some mathematical limits on the degree to which black-boxes can be truly, *a priori* explained. We also develop a computational method of fitting a model to cancer-growth data which is built around explainability first and foremost, excising nonlinearity and stochasticity where possible, and find that such a method can usefully direct and improve the efficiency of standard machine-learning techniques.

This paper is laid out as follows. We demonstrate first that it is impossible to truly account for the workings of even the simplest imaginable neural network, and then introduce an alternative “white-box” algorithm which can be used to quickly and reliably identify candidate equations for tumor growth. By using this algorithm, we can explainability identify the region of “parameter space”—and thus, in a sense, the “type” of tumor growth—appropriate to a particular disease. After this step has been applied, we are no longer “fighting blind,” and may leave more detailed fitting to neural networks. With this algorithm, we can both significantly reduce the time taken to fit patient-specific models of tumor growth and provide meaning to their parameters. The goal of explainability, then, does not have to slow down machine learning techniques, but can aid them in their search for appropriate models.

## 2. Materials and methods

### 2.1. Theory: The barriers to an analytically explainable neural network

In the following section we consider a idealized mathematical model of the graph neural network during its training process, without activation functions and with inductive biases sufficient to describe a physical system of *N* interacting objects. Each object within the system is represented by a node with two properties: the input “representation” value *x*_*i*_ (which may represent size, position, age, etc.), and the target property, whose true value is $y_{i}^{\prime }$. By considering many values of *x*_*i*_ and $y_{i}^{\prime }$, we aim to learn the relationship *y*_*i*_(*x*_1_, *x*_2_, … *x*_*N*_) between them; the goal is to produce a value of *y*_*i*_ as close as possible to $y_{i}^{\prime }$ on the training data. All properties in this model are one-dimensional for simplicity, but the mathematics behind it may easily be extended to multidimensional systems. Since we are describing observable quantities, we assume all properties are real.

A real graph neural network will use several layers of interconnected weights and activation functions to represent the relationship between any two objects; a separate computational layer will then learn how each object aggregates the information it receives from the rest of the system. In our model, we condense this operation into a single relationship, which we assume is of the form

(1) $$\begin{array}{l} y_{i}=\underset{jks}{\sum }w_{ijks}x_{i}^{k}x_{j}^{s} \end{array}$$

where 1 ≤ *j* ≤ *N* and *k, s* in principle range over all integers, so that we are considering the product of two Taylor expansions. In practice, because we cannot store infinite sums, we choose some combinations of *j, k, s* to describe our system. *w*_*ijks*_ are coefficients which we will adjust according to a loss function. This form encodes a number of physical assumptions: firstly, that the relationship *y*_*i*_ is continuous and differentiable; secondly, that it consists of a number of sub-relationships *y*_*ij*_, which combine additively; and thirdly, that the relationship *y*_*ij*_, which describes the effect of object *j* on object *i*, is dependent only on the properties of those nodes (i.e., on *x*_*i*_ and *x*_*j*_) and on no others, i.e., that each object interacts with every other object independently. Less obvious is that we are assuming the relationship is also *local*. Though we presumably have many values of *x*_*i*_ from different time-points, the relationship *y*_*i*_ depends on the value of the representations {*x*_*i*_} only at a single time-point. The system does not know about its previous states, and is assumed to have time-translational symmetry.

Having given the weights *w*_*ijks*_ some initial values, we now adjust them continuously according to their contribution to our loss function *L*, which describes the total “wrongness” of our current guesses:

(2) $$\begin{array}{l} \frac{\partial w_{ijks}}{\partial t}=-\alpha \frac{\partial L}{\partial w_{ijks}} \end{array}$$

We say the system has converged when no further adjustments remain to be made, i.e., when

(3) $$\begin{array}{l} \frac{\partial w_{ijks}}{\partial t}=\frac{\partial L}{\partial w_{ijks}}=0 \end{array}$$

for all weights.

What is the impact of our choice of loss function on the value of the relationships {*y*_*i*_} at convergence? We will use a slightly modified and generalized version of the loss function used by Cranmer et al. (2020), and include one “error” term designed to penalize divergence from target values, and another term, commonly referred to as the “regularization” term (Xu et al., 2015), designed to penalize the overall complexity of the system. The general form of our loss function is

(4) $$\begin{array}{l} L=\underset{i}{\sum }|y_{i}-y_{i}^{\prime }{|}^{m}+\beta \underset{ijks}{\sum }|w_{ijks}{|}^{n} \end{array}$$

Clearly, there are three adjustable hyperparameters here: the positive integers *m, n*, and the real and positive β. For the loss function closest to that used by Cranmer et al., m = 1 and n = 2, it can be shown that there are two possible values for convergence, depending on the value of the parameter β and the target value $y_{i}^{\prime }$. The proof is as follows and is based on a self-consistency argument.

We have at convergence

(5) $$\begin{array}{l} \frac{\partial L}{\partial w_{ijks}}=\frac{\partial |y_{i}-y_{i}^{\prime }|}{\partial y_{i}}\frac{\partial y_{i}}{\partial w_{ijks}}+2\beta w_{ijks}=0 \end{array}$$

and $\frac{\partial |y_{i}-y_{i}^{\prime }|}{\partial y_{i}}=1$ if $y_{i}\geq y_{i}^{\prime }$ and −1 otherwise, i.e., $\frac{\partial |y_{i}-y_{i}^{\prime }|}{\partial y_{i}}=\frac{y_{i}-y_{i}^{\prime }}{\left|y_{i}-y_{i}^{\prime }\right|}$, and $\frac{\partial y_{i}}{\partial w_{ijks}}=x_{i}^{k}x_{j}^{s}$, so we have convergence when

(6) $$\begin{array}{l} \frac{\partial L}{\partial w_{ijks}}=\frac{y_{i}-y_{i}^{\prime }}{\left|y_{i}-y_{i}^{\prime }\right|}x_{i}^{k}x_{j}^{s}+2\beta w_{ijks}=0 \end{array}$$

i.e., if $y_{i}\geq y_{i}^{\prime }$ we have $\left(y_{i}-y_{i}^{\prime }\right)\left(x_{i}^{k}x_{j}^{s}+2\beta w_{ijks}\right)=0$, and if $y_{i}<y_{i}^{\prime }$ we have $\left(y_{i}-y_{i}^{\prime }\right)\left(x_{i}^{k}x_{j}^{s}-2\beta w_{ijks}\right)=0$. So convergence at $y_{i}=y_{i}^{\prime }$ is possible for *any value of* *w*_*ijks*_.

For $y_{i}\geq y_{i}^{\prime }$ we also have a solution for convergence at $w_{ijks}=-\frac{x_{i}^{k}x_{j}^{s}}{2\beta }$. Now we can use our self-consistency argument, because *y*_*i*_ is defined by its contributing weights: thus this solution is possible if

(7) $$\begin{array}{l} y_{i}=\underset{jks}{\sum }w_{ijks}x_{i}^{k}x_{j}^{s}=\underset{jks}{\sum }-\frac{x_{i}^{2k}x_{j}^{2s}}{2\beta }\geq y_{i}^{\prime } \end{array}$$

which is to say we can have a different kind of convergence—what we will call “information-free” convergence—at $y_{i}=\underset{jks}{\sum }-\frac{x_{i}^{2k}x_{j}^{2s}}{2\beta }$ provided that $y_{i}^{\prime }\leq \underset{jks}{\sum }-\frac{x_{i}^{2k}x_{j}^{2s}}{2\beta }\leq 0$ for all *j, k, s* combinations used to describe our system. An identical argument for the $y_{i}<y_{i}^{\prime }$ case allows such information-free convergence at $y_{i}=\underset{jks}{\sum }\frac{x_{i}^{2k}x_{j}^{2s}}{2\beta }$ if $y_{i}^{\prime }>\underset{jks}{\sum }\frac{x_{i}^{2k}x_{j}^{2s}}{2\beta }\geq 0$.

In summary, then, if $|y_{i}^{\prime }|\leq \frac{\underset{jks}{\sum }x_{i}^{2k}x_{j}^{2s}}{2\beta }$, then convergence is only reached at $y_{i}=y_{i}^{\prime }$ for all *i*, with no restriction placed upon the weights *w*_*ijks*_. We refer to this as “absolute convergence.” If any target value falls outside of those restrictions (i.e., $|y_{i}^{\prime }|>\frac{\underset{jks}{\sum }x_{i}^{2k}x_{j}^{2s}}{2\beta }$ for any *i*), then in addition to absolute convergence, we have a second possibility: that relationship *y*_*i*_ may converge at $|y_{i}^{\prime }|=\frac{\underset{jks}{\sum }x_{i}^{2k}x_{j}^{2s}}{2\beta }$. This is, of course, a completely meaningless value, independent of $y_{i}^{\prime }$ and indeed of any individual property of the node *i*. This is why we refer to this possibility as “information-free” (I-F) convergence. It, too, places no restriction on the value of the weights; the system is not guaranteed to be made any simpler, which of course would be little reassurance, given that the relationship it describes is essentially “random.”

From this, we see that we can mitigate the possibility of I-F convergence by setting


$$\beta \ll \frac{\sum_{jks}x_{i}^{2k}x_{j}^{2s}}{2}$$

thus widening the range of values of $y_{i}^{\prime }$ within which only absolute convergence is possible; and I-F convergence is avoided entirely by setting β = 0. What, then, is the point of having a regularization term in this model at all, if not for its original intended purpose of making the result ‘simpler’? The answer is that it makes convergence *faster*. The speed of convergence of this loss function is determined by

(8) $$\begin{array}{l} \frac{\partial L}{\partial t}=\underset{ijks}{\sum }\frac{\partial L}{\partial w_{ijks}}\frac{\partial w_{ijks}}{\partial t}=-\alpha \underset{ijks}{\sum }{\left(\frac{\partial L}{\partial w_{ijks}}\right)}^{2} \end{array}$$

as the weights are adjusted according to $\frac{\partial w_{ijks}}{\partial t}=-\alpha \frac{\partial L}{\partial w_{ijks}}$ within our model. In the limit β → 0, $\frac{\partial L}{\partial t}\to -\underset{i}{\sum }\alpha {\left(\frac{y_{i}-y_{i}^{\prime }}{\left|y_{i}-y_{i}^{\prime }\right|}\right)}^{2}=-\underset{i}{\sum }\alpha$, i.e., decline is constant and at a rate proportional to α and to the number of objects in the system. Conversely, in the limit β → ∞, $L\to \beta \underset{ijks}{\sum }w_{ijks}^{2}$ and $\frac{\partial L}{\partial t}\to -\alpha \underset{ijks}{\sum }4\beta^{2}w_{ijks}^{2}=-4\alpha \beta L$, so $L=L_{0}e^{-4\alpha \beta t}$, and convergence is exponential with time.

This example is simple but illustrative: even within this toy model, the loss function does not have an intuitive effect on convergence values. For the general even-power case *m* = *n*, it can be shown similarly (proof in Appendix, Section 1) that at convergence,

(9) $$\begin{array}{l} y_{i}=\frac{y_{i}^{\prime }}{1+\frac{\beta^{\frac{1}{n-1}}}{\sum_{jks}{\left(x_{i}^{k}x_{j}^{s}\right)}^{\frac{n}{n-1}}}} \end{array}$$

with a corresponding equation for weights. We see now the *scale* on which the value of β should be considered: what governs the final output guess is the ratio $\frac{\beta^{\frac{1}{n-1}}}{\underset{jks}{\sum }{\left(x_{i}^{k}x_{j}^{s}\right)}^{\frac{n}{n-1}}}$. In the limit of large *n*, since *n* is even, the denominator tends to $\underset{jks}{\sum }|x_{i}^{k}x_{j}^{s}|$, which we may think of as the “sum of the total information in the subsystem *i*.” In that limit, the effect of increasing β is blunted by the fact that the relevant quantity is its *n* − 1-th root. In the limit $\beta^{\frac{1}{n-1}}\ll \underset{jks}{\sum }{\left(x_{i}^{k}x_{j}^{s}\right)}^{\frac{n}{n-1}}$, we recover absolute convergence, $y_{i}\to y_{i}^{\prime }$; in the limit $\beta^{\frac{1}{n-1}}\gg \sum_{jks}{\left(x_{i}^{k}x_{j}^{s}\right)}^{\frac{n}{n-1}}$, all weights in the subsystem *i* and the output guess *y*_*i*_ tend to zero. There is no possibility of information-free convergence to a non-zero value. This would seem, then, to be a much more appropriate choice of loss function. In Appendix (Section 1), we briefly discuss the general even-power *m, n* case, the case *m* = *n* = 2, and in Appendix (Section 3) we note the behavior of the more niche subcase of elastic regularization (Li et al., 2020).

Until now, we have discussed the effect of loss function hyperparameters on convergence values within an idealized linear model of a neural network. We will now attempt to incorporate the structure of a real neural network into our model—i.e., that of layers of nodes mediated by activation functions.

We model a simple two-layer network. We have two inputs, *x*_*i*_ and *x*_*j*_, which are fed into a hidden layer of nodes. The node indexed by *k* within this layer has output

(10) $$\begin{array}{l} v_{k}=a_{ki}x_{i}+a_{kj}x_{j}+b_{k} \end{array}$$

and our final guess *y* (we will drop the subscript *i* for the moment) is made by combining the outputs of the hidden layer, each fed through an activation function:

(11) $$\begin{array}{l} y=\underset{k}{\sum }c_{k}\phi \left(v_{k}\right)+\delta \end{array}$$

for the activation function used in the rectified linear unit, ϕ(*x*) = max(*x*, 0). We will use the loss function (4) with *m* = *n* = 2 which has bounded error, no information free-convergence, and whose error decays exponentially with time (proof in Appendix, Section 1). Here, it becomes:

(12) $$\begin{array}{l} L={\left(y-y^{\prime }\right)}^{2}+\beta \underset{k}{\sum }a_{ki}^{2}+a_{kj}^{2}+b_{k}^{2}+c_{k}^{2}+\delta^{2} \end{array}$$

At convergence we obtain a self-consistency equation for the node outputs *v*_*k*_:

(13) $$\begin{array}{l} v_{k}=\frac{{\left(y-y^{{}^{\prime }}\right)}^{2}}{\beta^{2}}\left(x_{i}^{2}+x_{j}^{2}+1\right)\phi \left(v_{k}\right) \end{array}$$

This imposes either *v*_*k*_ = 0 or, for *v*_*k*_ > 0, $|y-y^{\prime }|=\frac{\beta }{\sqrt{x_{i}^{2}+x_{j}^{2}+1}}$, i.e. a minimum error at convergence that tends to infinity with β. Further, constructing the guess *y* directly from our convergence equations for *c*_*k*_, we obtain the result (full proof in Appendix, Section 2) that for target guesses within the range

(14) $$\begin{array}{l} |y^{\prime }|<\frac{\beta +1}{\sqrt{x_{i}^{2}+x_{j}^{2}+1}} \end{array}$$

convergence is *impossible*. Even taking the limit β → 0 cannot eliminate this effect entirely, and the range to which it applies widens without bound as β → ∞. This is worth restating: in the simplest realistic model of a neural network that incorporates activation functions, there are ranges of representations and target values—unalterable input data—for which convergence becomes mathematically impossible, and the learning process will never terminate. In practice, of course, real networks do not converge only when the gradient of the loss function with respect for each weight is precisely zero: we will consider the network converged when the magnitude of the gradient of each weight has reached some small value ε. From the standpoint of the white-box modeler, unfortunately, this is hardly any better. If there is some large number *N*_*w*_ of weights in the system, then all we can say with certainty is that convergence occurs somewhere within a high-dimensional hyperspace of volume ${\left(2\varepsilon \right)}^{N_{w}}$, which leaves us with a very large number of possible configurations of the system, of which the “correct” one will be chosen stochastically. The system has become unexplainable once again.

How do we build an algorithm which does not run into these analytical difficulties, and has explainability as its central goal? If our aim is to construct a procedure that can correctly analyze a physical system, whose workings are completely mathematically transparent, and which is guaranteed to converge, our analysis above suggests we should move away from the realm of gradient descent and nonlinear units entirely, and begin from first principles. We follow this approach in the section below.

### 2.2. A white-box algorithm for characterizing tumor growth

Suppose that we have chosen some *i, j, k, s* combinations to describe our system, so that we assume relationships are of the form

(15) $$\begin{array}{l} y_{i}=\underset{jks}{\sum }w_{ijks}x_{i}^{k}x_{j}^{s}=\underset{m}{\sum }f_{im}z_{im} \end{array}$$

where we have condensed the weights *w*_*ijks*_ and terms $x_{i}^{k}x_{j}^{s}$ corresponding to the combinations {(*i, j, k, s*)} into *M*_*i*_ weights and terms *f*_*im*_, *z*_*im*_ corresponding to the object *i*. We will assume that we have samples of {*x*_*i*_} and $\left\{y_{i}^{\prime }\right\}$ for all objects, and for several configurations of the system. In all methods discussed above, we considered each timepoint independently; here we will combine them, and attempt to find the coefficients {*f*_*im*_} which produce the most accurate guesses across all timepoints and objects.

This raises two immediate concerns. One is a degrees-of-freedom issue: if we have *M*_*i*_ coefficients, then we can only guarantee accuracy at *M*_*i*_ time-points. However, if we actually have deduced the physical laws obeyed by our system, this should not matter; the correct relationships will hold at all time-points and not just the ones they were determined from. If we have chosen the wrong terms *z*_*im*_, our guess *y*_*i*_(*t*) will diverge from the target values $y_{i}^{\prime }\left(t\right)$ at times far away from those used to deduce the coefficients.

The second problem is one of “interpretability.” In theory, if we have *M*_*i*_ time-points, we have as many equations as variables, and we can determine our coefficients by simple linear algebra: if we define a vector ${\vec{Y}}_{i}^{\prime }$ of target values such that ${\left({\vec{Y}}_{i}^{\prime }\right)}_{j}=y_{i}^{\prime }\left(t_{j}\right)$ and a matrix $\underline{\underline{Z_{i}}}$ given by ${\left(\underline{\underline{Z_{i}}}\right)}_{jk}=z_{ij}\left(t_{k}\right)$, such that each row describes the value of a single term at each time-point, then our coefficients are straightforwardly given by solving the equation

(16) $$\begin{array}{l} {(\underline{\underline{Z_{i}}})}^{T}\cdot {\vec{F}}_{i}={\vec{Y}}_{i}^{\prime } \end{array}$$

for a vector ${\vec{F}}_{i}$ whose entries are the coefficients *f*_*im*_. However, this would involve the calculation of the matrix inverse of ${\left(\underline{\underline{Z_{i}}}\right)}^{T}$, which is both computationally fraught and analytically problematic. There is no easy general formula for the inverse of an *N*-by-*N* matrix, and so it is all but impossible to discern how the values of our chosen terms influence our final coefficients. Once we introduce the matrix inverse into our algorithm, it becomes a black box once again; it is impossible to construct, say, a useful differential equation in a single datapoint *z*_*ij*_(*t*_*k*_), if that term is incorporated into a matrix which is then inverted.

Instead we use Cramer's rule, first written down in 1,752 and of which there are many proofs widely available (including that in Brunetti, 2014). The coefficients are given by

$$\begin{array}{l} f_{im}=\frac{\left|\underline{\underline{S_{im}}}\right|}{\left|\underline{\underline{Z_{i}}}\right|} \end{array}$$

where square brackets indicate determinants and the matrix $\underline{\underline{S_{im}}}$ is defined by

(17) $$\begin{array}{l} {\left(\underline{\underline{S_{im}}}\right)}_{jk}=\left\{\begin{array}{c} z_{ij}\left(t_{k}\right),j\neq m;\\ y_{i}^{\prime }\left(t_{k}\right),j=m \end{array}\right\} \end{array}$$

This produces coefficients which exactly solve, for all chosen timepoints *t*_*k*_ (which we assume are randomly chosen from a dataset of possible observations),

(18) $$\begin{array}{l} y_{i}\left(t_{k}\right)=\underset{m}{\sum }f_{im}z_{im}\left(t_{k}\right)=y_{i}^{\prime }\left(t_{k}\right) \end{array}$$

The great benefit of this technique is that a determinant is linear in all values it involves. By avoiding the matrix inverse, we have ensured that the coefficient is differentiable in every element of data that contributes to it, and thus the effect of each piece of data on our conclusions is exactly quantifiable. This part of the algorithm is a completely “white box.”

The above procedure predicts the coefficients {*f*_*im*_} that best describe the system when presented with a set of terms {*z*_*im*_}; we must still develop a process for choosing between sets of terms. The simplest and best procedure is simply to try each possible set of terms sequentially and choose the set of terms {*z*_*im*_} which has the lowest error according to the loss function

(19) $$\begin{array}{l} L=\underset{i,t}{\sum }{\left(y_{i}\left(t\right)-y_{i}^{\prime }\left(t\right)\right)}^{2} \end{array}$$

where the sum is over all timepoints in the dataset, not simply the randomly-chosen timepoints used to deduce the coefficients. This is a straightforward way of determining the “goodness of fit” of our model, and has no hyperparameters, because we have eliminated the regularization term. Here, there is a much easier, more intuitive way of measuring the complexity of our system: the number of terms in our polynomial description, *M*_*i*_, which we control directly. We could make our loss function *L*_*n*_ instead of *L*_2_ for *n* ≥ 2 and even; clearly, this would have the effect of valuing a polynomial description with a large number of small errors over one with a small number of large errors, which may be desirable or not depending on the needs of the clinician.

We must, therefore, try each set of terms sequentially, however naive an approach that may initially seem. Any attempt to navigate the space of possible terms {*z*_*im*_} through stochastic gradient descent using the loss function *L* is doomed to failure, since we cannot move in infinitesimal increments through *z*_*im*_, but must jump between discrete sets of input data combinations, which may involve changes in value so large as to render gradients useless. Further, in order to determine the gradient of the loss function with respect to an input term *z*_*im*_, we must also consider its effect on the entire set of deduced coefficients {*f*_*im*_}, which will require two matrix determinant evaluations for every coefficient. At this point, the calculation of the gradient at each point becomes much more computationally expensive than simply calculating the loss for each set of terms, which is guaranteed to terminate, since the space it is exploring is finite. A brief analysis of cost, and an additional *generalizability* metric assessing the suitability of a particular description-length *M*_*i*_, is included in Appendix (Section 4).

### 2.3. Experiment: Fitting models of tumor growth

**Figure 1:**
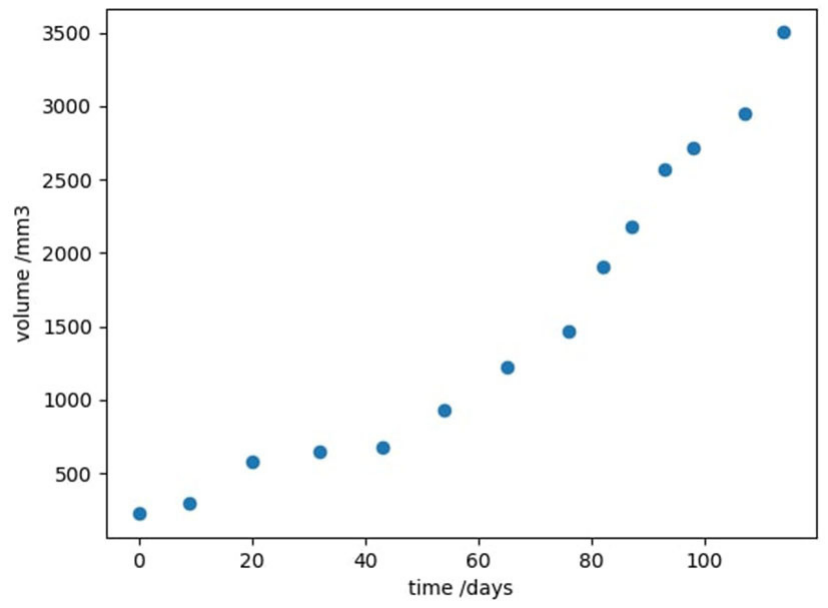
Experimental data showing the growth of tumor volume with time, in a mouse model of human breast cancer, taken from Kühleitner et al. (2019).

We now investigate the advantages of this algorithm when applied to real-world cancer data. For the remainder of this paper we will be following the work of Kühleitner et al. (2019). In this paper, the authors considered longitudinal time-series data of the growth of a tumor. Human breast cancer cells were injected into nude mice, and the resulting tumor volume *v*(*t*) was observed over 114 days, in a study by Worschech et al. (2009) (shown in Figure 1). Kühleitner et al. (2019) aimed to find the best parameter fit for a Bertalanffy-Pütter model from the observed tumor data; that is to fit the non-negative parameters *p, q, a, b* in the first-order differential equation


(20) $$\begin{array}{l} \frac{dv}{dt}=pv^{a}-qv^{b} \end{array}$$

The Bertalanffy-Pütter model (Ohnishi et al., 2014) is a general class of tumor-growth model which encompasses other, more specific tumor models, including the Verhulst model (Verhulst, 1838) (*a* = 1.0, *b* = 2.0) and the Gompertz model (*a* = 1.0, *b* > 1.0) (Gompertz, 1833). Per Kühleitner, it has been experimentally observed that tumors tend to shrink when they become very large; to ensure this behavior, only exponent-pairs *a* < *b* are considered. They were examined at intervals of 0.01, so that (*a* = 0.01*n, b* = *a* + 0.01*m*) for all valid non-negative integers *n, m* that placed (*a, b*) within the highlighted range. For every exponent-pair, the authors fitted the best coefficient-pair (*p, q*) through a painstaking process of stochastic gradient descent and simulation (simulated annealing), using the same *L*_2_ loss function (2), otherwise known as the sum of squared error (SSE), defined in our algorithm. Having chosen a trial pair (*p, q*), they solve the equation numerically over 144 days, sum the square of the errors, make a partially-stochastic adjustment to (*p, q*), and simulate again. Their final best fit was (*p* = 5 · 10^−4^, *q* = 5.6 · 10^−7^, *a* = 1.62, *b* = 2.44), obtained at a cost of roughly 1 week of CPU time. Our objective is to repeat this study by applying our algorithm to fit coefficients of the Bertalannfy-Pfutter model to this data using SSE as our loss function. We make these choices for ease of comparison, but the algorithm could in theory work with any differential-equation model and any loss function. If we were to use a stochastic differential equation (SDE), for example, we could generate a maximum likelihood function for a model defined by a given set of parameters, which would allow us to use likelihood-dependent loss functions, such as the Akaike and Bayesian Information Criteria.

## 3. Results

### 3.1. Identifying regions of good fit with real-world data

**Figure 2:**
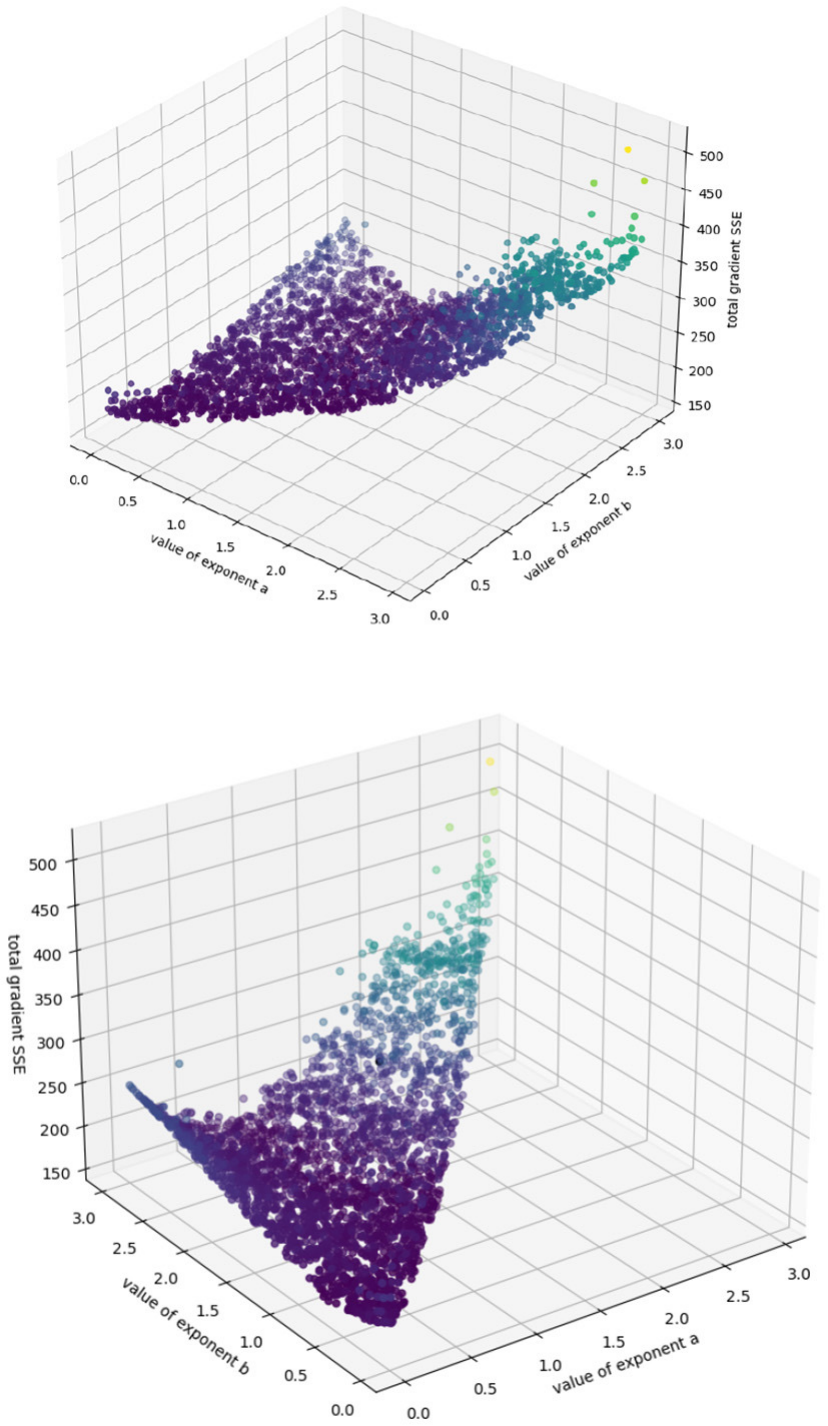
Sum of squared error from extrapolation from fitted (p, q) values for each exponent-pair value; color simply corresponds to height for highlighting purposes.

We have a single output guess, $y_{i}^{\prime }\left(t\right)=y^{\prime }\left(t\right)=\frac{dv}{dt}$, obtained using numpy.gradient's (Cranmer et al., 2020) first-order approximations at each timepoint instead of by precise and repeated simulation; we have a single input representation, *x*_*i*_(*t*) = *x*(*t*) = *v*(*t*), the observed tumor volume. Because we are fitting to a *known model* here instead of unknown dynamics, we do not need to involve the generalizability metric or decide between numbers of terms; instead we can simply try each (*a, b*) pair sequentially, deduce our coefficients (*p, q*) using Cramer's rule, and output an error *L* using the sum of the squares of the errors of the gradient at each timepoint according to that prediction. As we are only deducing two coefficients, we choose two timepoints at random; to make sure our predictions are an accurate reflection of the entire dataset, we repeat the procedure above 20 times for each (*a, b*) pair (to ensure that each datapoint has a 95% chance of being selected at least once), and choose the deduced coefficient pair (*p, q*) with the lowest error. We consider all exponent-pairs at 0.01 intervals where *a* < *b* ≤ 3.0, the highest value considered by Kühleitner et al. (2019). Our algorithm runs very quickly on a standard laptop (requiring just under seven minutes to terminate), and efficiently explores the space of possible parameters for the roughly 45,000 possible exponent pairs, returning the accuracy surface. Because we only have two coefficients to fit per exponent pair, this surface can be visualized in three dimensions (see Figure 2); this is an advantage of the Bertalanffy-Pütter model.

**Figure 3:**
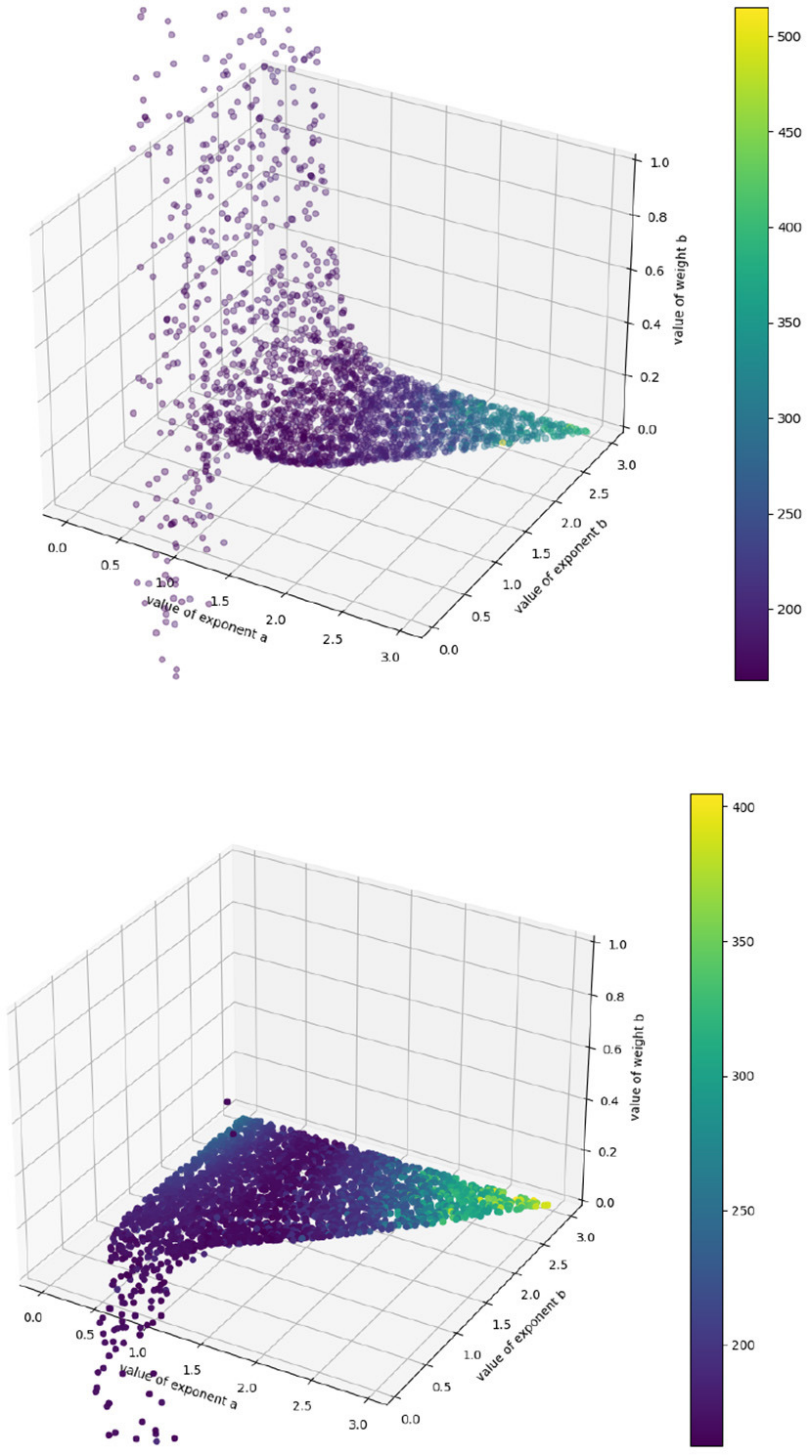
Fitted p (above) and q (below) values for each exponent-pair value. The colorbar corresponds to SSE—purple datapoints have lower error, yellow ones higher. The varying limits come from the fact that, to generate each plot, we randomly choose 2,500 points out of 45,000 to display.

Because our target values are imprecise approximations to the true growth rate, the algorithm cannot perfectly identify the actual accuracy minimum. However, this surface shows us intuitively how the model behaves in various regions of the (*a, b*) space. We can see, for example, that the model behaves asymptotically badly as the exponents increase past 2.5, and that no effort should be expended trying to identify (*p, q*) pairs there. We can also see a “valley” of low error in the center, which might be understood as a “region of good fit,” where exponent pairs generally describe the system well. We can also use this algorithm to identify regions of overfit, by plotting the best values of *p* and *q* obtained at each point in (*a, b*) space (see Figure 3).

We see that all regions where *a, b* < 1.0 should be ignored, as the coefficients “hit a wall” as soon as that threshold is passed: they become rapidly unstable (and, in the case of *q*, unphysically negative) with respect to small changes in exponent pairs, which suggests that region provides a poor model of the system, since any good mathematical model of a biological system should not be so acutely sensitive to small changes in its terms. This allows us to narrow down the promising region of (*a, b*) in space to the section of the valley where *a, b* > 1.0, and we can explore that region further using precise simulation to identify the best coefficient-pair (*p, q*). Further, we have a good idea of where those coefficients should lie: for the authors' final best exponent pair (*a* = 1.62, *b* = 2.44) we obtain (*p* = 3 · 10^−4^, *q* = 3 · 10^−7^) to their (*p* = 5 · 10^−4^, *q* = 5.6 · 10^−7^), which is remarkably close given that their gradients are derived from careful simulation and ours from crude first-order approximation. We have narrowed down the space of possible hyperparameters by several orders of magnitude in a matter of minutes; what remains can then be explored more precisely.

### 3.2. Recovering parameters from synthetic data

We can test the algorithm's accuracy further by using this surface to identify trial parameters, generate synthetic data using those parameters, and using the algorithm to retrieve them. We assume that every set of (*a, b, p, q*) parameters with SSE smaller than that of the “official” Kühleitner solution is biologically realistic, as it fits the tumor growth trajectory at least as closely. We limit ourselves to the region *a, b* > 1.0 and obtain about 5,000 possible sets of parameters, from which we select 1,000 at random. Using the initial tumor volume as our starting point, for every chosen (*a, b, p, q*) we extrapolate forward according to equation (20).

**Figure 4:**
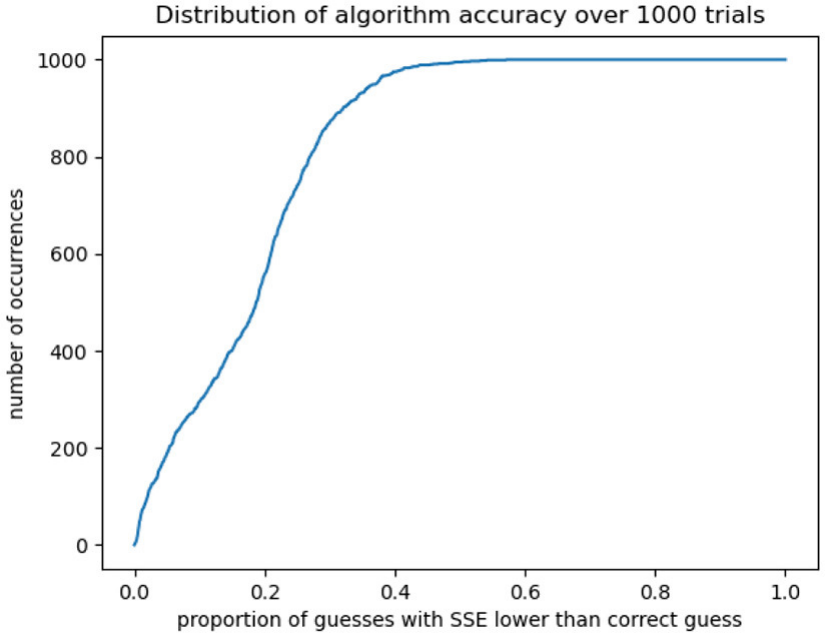
For 1,000 synthetically generated test cases, we calculate whether the correct exponent-pair occurs within the lowest-SSE “x” percent of the space. In most cases the algorithm can isolate roughly two-fifths of the original space which may then be explored in more detail for a closer-fitting solution.

We then take the tumor volumes at the same timepoints as the original data, to mimic its sparsity. We generate an accuracy surface for each trajectory according to the procedure above (This process took roughly 36 h using the University College London DPS machines). For each “synthetic tumor,” we denote the exponent-pair used to generate it as (*a*_∗_, *b*_∗_), and calculate the fraction of the parameter space 1.0 < = *a, b* =< 3.0 with an assigned SSE lower than that calculated for (*a*_∗_, *b*_∗_). This gives us a neat metric for the degree to which the algorithm “narrows down” the parameter space, depending on how confident the modeler wishes to be that the “correct” parameter values—insofar as any biological system can be said to have a single correct set of underlying parameters—lies within the identified region. Our results are shown in Figure 4. For 999 out of 1,000 trajectories, (*a*_∗_, *b*_∗_) has an SSE higher than 57% of the parameter space; for 990 trajectories, we can narrow down to 46% of the space, for 950, to 37%; for 900, to 32%; and for 800 to 27%. We see a “threshold effect,” demonstrated below: in the vast majority of cases the space can be narrowed down to roughly two-fifths of its original area.

### 3.3. The effect of noise on algorithmic efficacy

**Figure 5:**
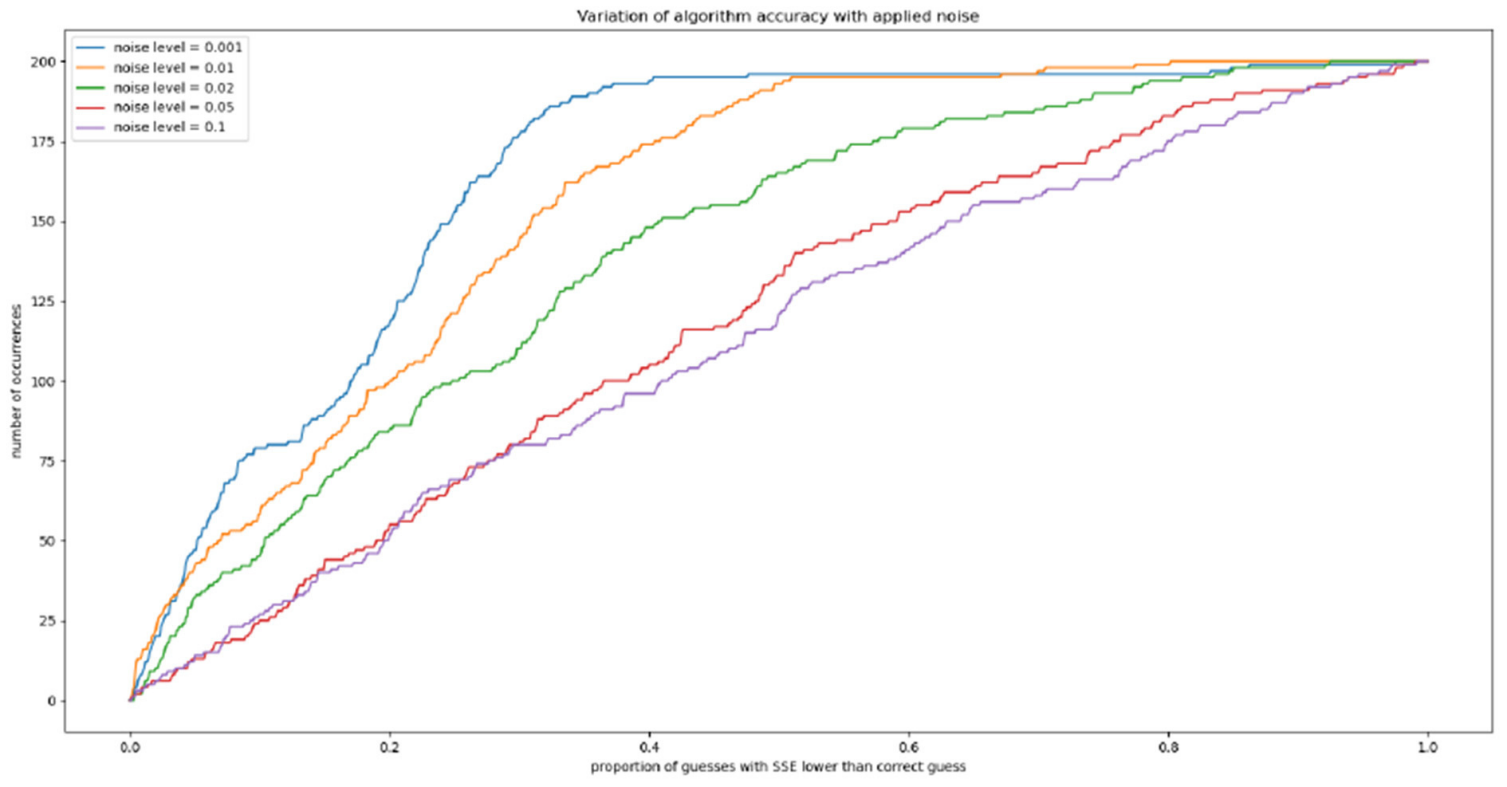
The effect of randomly-generated noise on algorithm accuracy.

We can also explore the effect of noise on this accuracy, by separating our 1,000 trajectories into five groups of 200 and injecting random noise at each timepoint. For a noise level of 0.01, for example, at each timepoint a random fraction of the tumor volume between 1 and −1% is drawn from a normal distribution and added to the tumor volume. Gradients are then computed and the algorithm is run as previously; we again calculate the proportion of the parameter space with an SSE lower than that assigned to the correct exponents (*a*_∗_, *b*_∗_). Our results are shown in Figure 5. We see that the “thresholding” effect, by which the correct parameters can be narrowed down to a certain proportion of the space with near-certainty, holds up to a noise level of roughly 0.02.

## 4. Discussion

By attempting to build an algorithm that can interpretably explain the unknown dynamics of an interacting system, we have found an approach that can quickly and easily explore the space of parameters of a differential equation which incorporates a variety of models of tumor growth. On synthetic tumor-growth data, the algorithm can reliably (with a probability of 95%) more than halve the region of parameter space that requires finer searching using less rigorous, more computationally expensive machine learning methods. There is good reason to think the algorithm can be usefully applied to more general models of cancer growth, so long as there are enough datapoints that the compromise of first-order gradient estimation can be safely made. In fact, above approach does not require the underlying equation to be first-order, or indeed to be a differential equation at all; it works for any form, any number of terms, and any number of objects. It provides a first-approximation to the behavior of the system, without the expense of simulation, and it does so without nonlinearity or the use of hyperparameters. It can therefore be applied to a variety of contexts, medical and otherwise.

An important aspect of the above procedure, at least as it applies to cancer modeling, is that it identifies not simply one good fit to the equation—as stochastic gradient descent does—but instead identifies several thousand candidate equations and ranks them by “goodness of fit.” This is particularly useful to us because a tumor is not a purely deterministic or mathematical object: it does not obey a single equation for all time, and its behavior is likely best modeled as a combination of, or a movement through, the candidate equations suggested by the algorithm. The ability to *narrow down* the space of model parameters to describe a particular tumor—perhaps successively, through more and more granular exploration—will be of use to clinicians trying to classify and predict the behavior of cancers. Even leaving aside explainability considerations, our algorithm can more than halve the space which must be explored to fit parameters to the tumor using stochastic gradient descent, which is a vital efficiency gain when trying to provide personalized predictions at scale. There are a wide range of complex interacting-differential-equation models of cancer growth to which this algorithm might usefully be applied (for instance, Nave, 2020; Hori et al., 2021; Mascheroni et al., 2021; Nave and Elbaz, 2021), although the algorithm could, again, in principle be used to describe any dynamical system.

**Figure 6:**
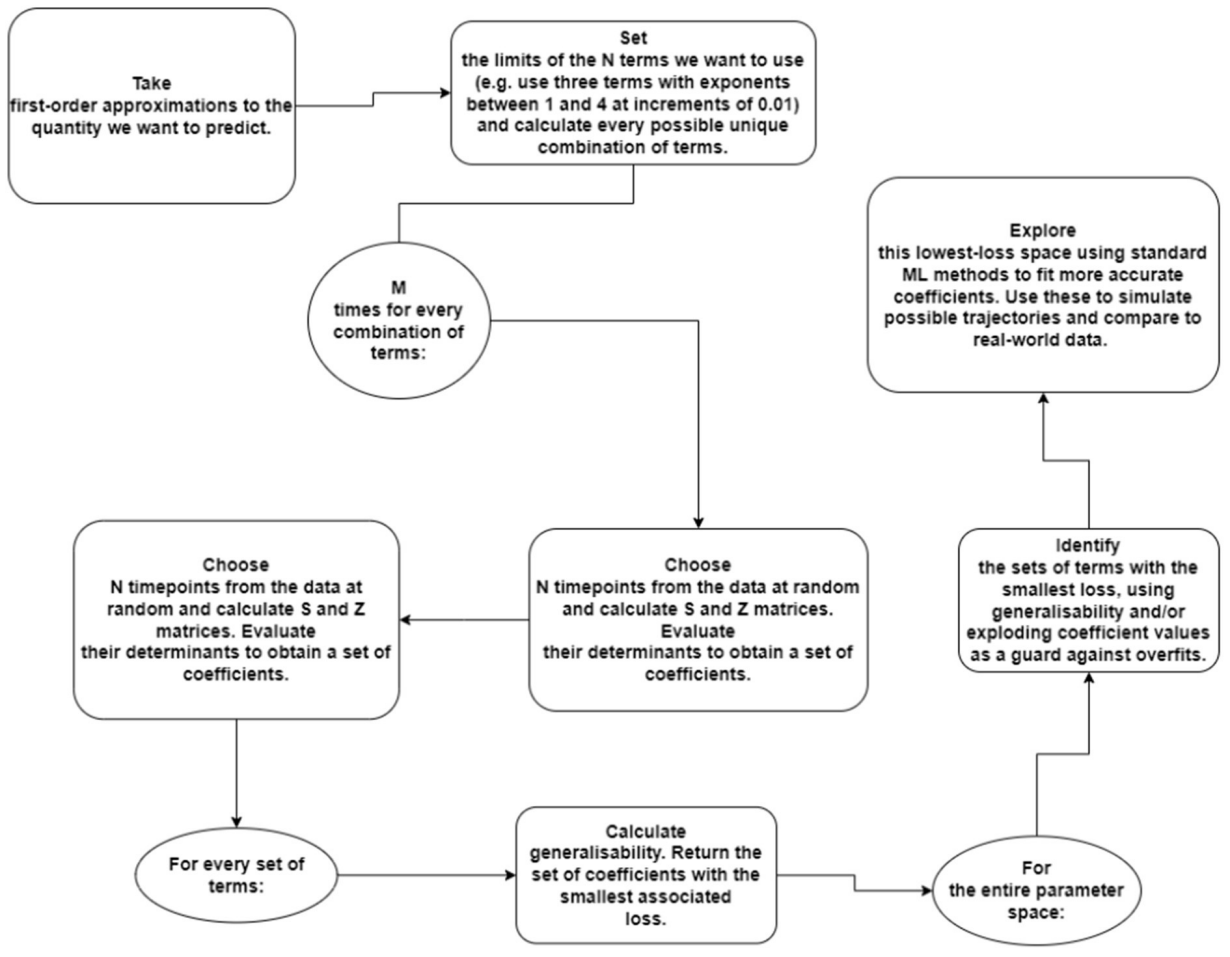
Diagram of the algorithmic procedure for the preliminary investigation of physical systems. In this example, we are using an N-term polynomial with M trials each.

In addition to this, across patients, the accuracy surface may provide a useful tool for characterizing particular kinds of cancer, or the effects of certain treatments. It may be that further study reveals that there is a link between the best regions of (*a, b*) space to describe a tumor and some aspect of its growth or behavior. The ability to associate a set of best-fit (*p, q, a, b*) parameters to a particular tumor also suggests the possibility of new set of survival metrics, which may correlate directly the prognosis of human patients. This merits further investigation. A full diagram of the procedure is included in Figure 6.

A technical aspect of the algorithm worth drawing attention to is its susceptibility to underflow errors, which arises from its calculation of the ratio of two determinants. This is not an issue in any of the cases discussed above, but rapidly compromises any current attempt to apply the algorithm to large systems or to use many terms. If we have *M* terms in our description, for example, each of the order 10^−*n*^, then the coefficients will be ratios of two numbers of order 10^−*nM*^. Given that standard Python floating-point precision cannot accurately represent numbers smaller than about 10^−39^ (Rajaraman, 2016), neither *n* nor *M* have to become very large before we run into accuracy issues. Further work could implement the algorithm using an arbitrary-precision arithmetic program designed specifically to compute matrix determinants, such as Arb (Johansson, 2017). The algorithm also requires its input data to be sufficiently detailed that the compromise of first-order gradient approximation is worth making. On datasets such as that attached to Laleh et al. (2022), where most trajectories are composed of six or fewer datapoints, attempts to fit exponents result in flat, highly noisy surfaces with no significant curvature. Mouse or *in vitro* models, which can be monitored more or less continuously without the need for painful and invasive scans on human subjects, are our likeliest sources of useful data. However, as scanning methods become more advanced over the next decade (Rockne et al., 2019)—less invasive, less painful, and cheaper to perform regularly on human patients—tumor-volume trajectories will become denser and more amenable to mathematical analysis, and the context in which this algorithm is useful will move from the experimental to the clinical.

## 5. Conclusion

This paper describes an interpretable method for quickly surveying the parameter space of various differential-equation models. It is precisely the complexity and nonlinearity of neural networks which make them so useful in problems of classification or recognition, but when human lives are at stake, it is important to develop methods of generating predictions and informing treatments that are built around explainability and *a priori* justification. Clinicians and patients must understand as much as possible where their information is coming from, and mathematical models derived from computational methods must be rigorous. Moreover, as our work on Kühleitner et al. (2019) shows, it is not even clear that immediately resorting to machine learning makes anything *faster*. Slow brute-force adjustment is an inefficient approach when a straightforward algorithm can narrow down the space of possible parameters, and suggest thousands of candidate equations, in a matter of minutes. In addition to the detailed machine learning work currently being done in the field of mathematical oncology (see for instance Bekisz and Geris, 2020), a different approach is needed—the unification of mathematics and machine learning to create a rigorous, explainable justification for the directions in which neural networks should be sent. We suggest the use of this first-order “exploration algorithm” as a first line of defense when modeling the behavior of cancer, to provide an initial understanding of the behavior of a model across its parameter space and significantly reduce the time taken to fit predictive equations. A return to first principles in cancer modeling may yield significant optimization.

## Data availability statement

The original contributions presented in the study are included in the article/Supplementary material, further inquiries can be directed to the corresponding author/s. All code used in the production of these results is available on request.

## Author contributions

HC developed proofs and experiments and drafted the paper under the close supervision of PL and with the advice of HA, both of whom also edited the paper. All authors contributed to the article and approved the submitted version.

## Funding

HC was supported by a grant from the Engineering and Physical Sciences Research Council, reference EP/W523835/1. The University College London Mathematics Department DPS machines were used to conduct some of the computational experiments in this paper. PL acknowledges funding from HORIZON-EIC (project number 101058004): Chemometric histopathology via coherent Raman imaging for precision medicine.

## Conflict of interest

The authors declare that the research was conducted in the absence of any commercial or financial relationships that could be construed as a potential conflict of interest.

## Publisher's note

All claims expressed in this article are solely those of the authors and do not necessarily represent those of their affiliated organizations, or those of the publisher, the editors and the reviewers. Any product that may be evaluated in this article, or claim that may be made by its manufacturer, is not guaranteed or endorsed by the publisher.
