# Supplementary material for: A novel interpretable machine learning algorithm to identify optimal parameter space for cancer growth
Source: Front Big Data. 2022 Sep 12;5:941451. doi: 10.3389/fdata.2022.941451 (PMC9510846; doi:10.3389/fdata.2022.941451)
Supplement: Supplementary file 1 [file Data_Sheet_1.pdf]

Appendices

Coggan *et. al.*

August 2022

1 Appendices

1.1 Proof: The Even-Power Loss Function Modulates Weights Continuously

Now we consider the equation (4) case

$$L = \sum_i (y_i - y_i')^m + \beta \sum_{ijks} w_{ijks}^n$$

for m, n both even. In this case, the convergence requirement $\frac{\partial L}{\partial w_{ijks}} = 0$ sets

$$m x_i^k x_j^s (y_i - y_i')^{m-1} + \beta n w_{ijks}^{n-1} = 0 \tag{1}$$

and therefore

$$m x_i^k x_j^s (y_i - y_i')^{m-1} + \beta n w_{ijks}^{n-1} = 0 \tag{2}$$

(We do not need to worry about encountering complex numbers here; since we stipulated n must be even, $n-1$ must be odd, and the $n-1$th root of a negative number is real and negative.) Again we use a self-consistency argument to relate w_{ijks} and y_i:

$$y_i = \sum_{jks} w_{ijks} x_i^k x_j^s \tag{3}$$

$$= -(\frac{m}{n\beta})^{\frac{1}{n-1}} (y_i - y_i')^{\frac{m-1}{n-1}} \sum_{jks} (x_i^k x_j^s)^{\frac{n}{n-1}} \tag{4}$$

$$\tag{5}$$

And therefore we reach

$$(y_i)^{\frac{n-1}{m-1}} + \left(\frac{m}{n\beta}\right)^{\frac{1}{m-1}} \left(\sum_{jks} (x_i^k x_j^s)^{\frac{n}{n-1}}\right)^{\frac{n-1}{m-1}} (y_i - y_i') = 0 \tag{6}$$

In the case $m = n = 2$, we have

$$w_{ijks} = -\frac{x_i^k x_j^s}{\beta}(y_i - y_i') \tag{7}$$

and so

$$y_i = -(y_i - y_i') \sum_{jks} \frac{x_i^{2k} x_j^{2s}}{\beta} = \frac{y_i'}{1 + \frac{\beta}{\sum_{jks} x_i^{2k} x_j^{2s}}} \tag{8}$$

In the limit $\beta \to 0$, $L \to \sum_i (y_i - y_i')^2$ and $\frac{\partial L}{\partial t} = -\alpha \sum_{ijks} (\frac{\partial L}{\partial w_{ijks}})^2 \to -4\alpha \sum_i (y_i - y_i')^2 = -4\alpha L$, so $L = L_0 e^{-4\alpha t}$; in the limit $\beta \to \infty$, $L \to \beta \sum_{ijks} w_{ijks}^2$ and $\frac{\partial L}{\partial t} \to -\alpha \sum_{ijks} 4\beta^2 w_{ijks}^2 = -4\alpha\beta L$, so $L = L_0 e^{-4\alpha\beta t}$, and the loss decays exponentially in *both limits*, though faster with increasing β, the trade-off for that, of course, is that y_i is decreased away from y_i'. We can also say that the error produced is 'bounded', since we have $0 < y_i <= y_i'$ for $y_i' > 0$ and $0 > y_i >= y_i'$ for $y_i' < 0$.

In the more general case where $m = n$, the convergence equation becomes

$$y_i + \beta^{-\frac{1}{n-1}} \left(\sum_{jks} (x_i^k x_j^s)^{\frac{n}{n-1}}\right) (y_i - y_i') = 0 \tag{9}$$

and therefore we have equation (9).

$$y_i = \frac{y_i'}{1 + \frac{\beta^{\frac{1}{n-1}}}{\sum_{jks} (x_i^k x_j^s)^{\frac{n}{n-1}}}}$$

1.2 Proof: Activation Functions Prevent Convergence Entirely For Some Input Ranges

Now we consider the inclusion of activation functions. To reiterate: we have two inputs, x_i and x_j, which are fed into a hidden layer of nodes. The node indexed by k within this layer has output $v_k = a_{ki}x_i + a_{kj}x_j + b_k$, and our final guess y is made by combining the outputs of the hidden layer, each fed through an activation function, such that $y = \sum_k c_k\phi(v_k) + \delta$. Our loss function is $L = (y - y')^2 + \beta\sum_k a_{ki}^2 + a_{kj}^2 + b_k^2 + c_k^2 + \delta^2$. Taking the derivative with respect to each weight and setting them to zero at convergence, we have

$$a_{ki} = -\frac{(y-y')c_k x_i \theta(\nu_k)}{\beta} \tag{10}$$

$$a_{kj} = -\frac{(y-y')c_k x_j \theta(\nu_k)}{\beta} \tag{11}$$

$$b_k = -\frac{(y-y')c_k \theta(\nu_k)}{\beta} \tag{12}$$

$$c_k = -\frac{(y-y')\phi(\nu_k)}{\beta} \tag{13}$$

$$\delta = -\frac{(y-y')}{\beta} \tag{14}$$

$$\tag{15}$$

where $\theta(\nu_k)$ denotes the Heaviside step function. Substituting these values into our expression for ν_k, we obtain

$$v_k = \frac{\left(y - y'\right)^2}{\beta^2}\left(x_i^2 + x_j^2 + 1\right)\theta(\nu_k)\phi(v_k) \tag{16}$$

$$= \frac{\left(y - y'\right)^2}{\beta^2}\left(x_i^2 + x_j^2 + 1\right)\phi(v_k) \tag{17}$$

since the form of the activation function absorbs the Heaviside function. This has two solutions: either $v_k = 0$ or, for $v_k > 0$, $|y - y'| = \frac{\beta}{\sqrt{x_i^2 + x_j^2 + 1}}$. Here we return to self-consistency arguments. Using the definition $y = \sum_k c_k\phi(v_k) + \delta$, we substitute in convergence values to obtain $y = -\frac{y-y'}{\beta}(1 + \sum_k v_k^2)$. If we choose the positive configuration $y - y' = \frac{\beta}{\sqrt{x_i^2+x_j^2+1}}$, then another substitution gives $y = -\frac{1}{\sqrt{x_i^2+x_j^2+1}}(1 + \sum_k v_k^2)$.

Now we have $y - y' = -\frac{1}{\sqrt{x_i^2+x_j^2+1}}(1 + \sum_k v_k^2) - y' = \frac{\beta}{\sqrt{x_i^2+x_j^2+1}}$, which rearranges to give us $\sum_k v_k^2 = -(1 + \beta + y'\sqrt{x_i^2 + x_j^2 + 1})$. But, of course, since all quantities are real, $\sum_k v_k^2 > 0$ (assuming that at least one node has a nonzero output); so to avoid a contradiction, we must have $y' < -\frac{\beta+1}{\sqrt{x_i^2+x_j^2+1}}$. Returning to our expression for $|y - y'|$ and choosing the negative configuration $y - y' = -\frac{\beta}{\sqrt{x_i^2+x_j^2+1}}$, we follow the same argument to find the requirement $y' > \frac{\beta+1}{\sqrt{x_i^2+x_j^2+1}}$. So, for values of y' in the range

$$|y'| < \frac{\beta + 1}{\sqrt{x_i^2 + x_j^2 + 1}}$$

we cannot have convergence if at least one node has a nonzero output (which it must in order for the network to be 'working' at all).

1.3 Aside: Hyperparameters in Elastic Regularisation Can Have Unbounded Effects

Now we turn to the case of elastic regularisation, given by the loss function

$$L = \sum_i \left(y_i - y_i'\right)^2 + \sum_{ijks}\beta_1|w_{ijks}| + \beta_2 w_{ijks}^2. \tag{18}$$

Taking the derivative with respect to any given weight, we have

$$\frac{\partial L}{\partial w_{ijks}} = 2(y_i - y_i')x_i^k x_j^s + \beta_1\frac{|w_{ijks}|}{w_{ijks}} + 2\beta_2 w_{ijks} \tag{19}$$

If w_{ijks} is non-negative, we have at convergence

$$w_{ijks} = -\frac{(y_i - y_i')x_i^k x_j^s}{\beta_2} - \frac{\beta_1}{2\beta_2} \geq 0 \tag{20}$$

We assume that, by construction, $\beta_1, \beta_2 \geq 0$, since if one or both were negative the regularisation term

would either be minimised as $w_{ijks} \to \infty$ or have a minimum for some $w_{ijks} > 0$. So to place a limit on y_i, we must consider two further sub-cases: where the term $x_i^k x_j^s > 0$ and so we have

$$y_i \leq y_i' - \frac{\beta_1}{2x_i^k x_j^s} \tag{21}$$

or, if $x_i^k x_j^s < 0$, we have

$$y_i \geq y_i' - \frac{\beta_1}{2x_i^k x_j^s} \tag{22}$$

(If $x_i^k x_j^s = 0$, no associated weight can exist; firstly for the practical reason that the term adds nothing to our description of the system and so we should not include it, and secondly because we then either have $w_{ijks} = -\frac{\beta_1}{2\beta_2} \geq 0$ or $w_{ijks} = \frac{\beta_1}{2\beta_2} < 0$, both of which are contradictions since $\beta_1, \beta_2 \geq 0$.)

An identical series of arguments leads us to the conclusion that, if $w_{ijks} < 0$ and $x_i^k x_j^s > 0$, we must have $y_i > y_i' + \frac{\beta_1}{2x_i^k x_j^s}$, and if $w_{ijks} \geq 0$ and $x_i^k x_j^s < 0$, we must have $y_i \leq y_i' + \frac{\beta_1}{2x_i^k x_j^s}$.

It is worth standing back here and considering what we have found. For every weight w_{ijks} and term $x_i^k x_j^s$ contributing to y_i, one of the above conditions must apply, depending on the relative sign of the descriptors. But these are not independent conditions: two of them, for the weight and term having the same sign, fix $y_i > y_i'$, and the other two, for weight and term having the opposite sign, fix $y_i < y_i'$. So we must identify two 'regimes' for the value of y_i. In one, $y_i < y_i'$, and every weight contributing to it has the same sign as its corresponding term; then $w_{ijks} x_i^k x_j^s = -\frac{(y_i - y_i')x_i^{2k} x_j^{2s}}{\beta_2} - \frac{\beta_1 |x_i^k x_j^s|}{2\beta_2}$ and so

$$y_i = -\sum_{jks} \frac{(y_i - y_i')x_i^{2k} x_j^{2s}}{\beta_2} - \sum_{jks} \frac{\beta_1 \left|x_i^k x_j^s\right|}{2\beta_2} \tag{23}$$

which rearranges to give

$$y_i = \frac{y_i' - \frac{\beta_1 \sum_{jks} |x_i^k x_j^s|}{2 \sum_{jks} x_i^{2k} x_j^{2s}}}{1 + \frac{\beta_2}{\sum_{jks} x_i^{2k} x_j^{2s}}} \tag{24}$$

Similarly, there is a solution in the 'opposite-sign scheme' where $y_i < y_i'$:

$$y_i = \frac{y_i' + \frac{\beta_1 \sum_{jks} |x_i^k x_j^s|}{2 \sum_{jks} x_i^{2k} x_j^{2s}}}{1 + \frac{\beta_2}{\sum_{jks} x_i^{2k} x_j^{2s}}} \tag{25}$$

Here, β_2 plays the role of the hyperparameter β in the m=2, n=2 single-regulariser loss function discussed above. β_1, however, has a very different effect. Instead of pushing the ratio $\frac{y_i}{y_i'}$ between zero and unity, it defines a 'distance of closest approach', a minimum difference between y_i and y_i' which cannot be overcome by setting β_2 to a particular value. Unlike β_2; it has an *unbounded* effect on y_i; as $\beta_1 \to \infty$, y_i is pushed away from y_i' and towards an infinity. Because it can force the output guesses, and therefore the weights, to be of unlimited magnitude, it does not follow that β_1 makes the system uniformly 'simpler' by any definition. There is also a stronger condition on convergence: for the solution where $y_i < y_i'$, then $y_i \leq y_i' - \frac{\beta_1}{2|x_i^k x_j^s|}$ for all contributing terms, and for $y_i \geq y_i'$, we must have $y_i \geq y_i' + \frac{\beta_1}{2|x_i^k x_j^s|}$ for all terms; so the mere inclusion of terms with very small magnitude can wreck our accuracy at convergence.

1.4 Aside: Cost Analysis and Suitability Metrics for White-Box Algorithm

If we decide to represent our system using a polynomial of M_i terms, and we allow the exponents k_{im} and s_{im} in $z_{im} = x_i^{k_{im}} x_{j_{im}}^{s_{im}}$ to take each of K possible values, then we have $Q_i = \frac{(NK^2)!}{M_i!(NK^2 - M_i)!}$ possible terms for the description of output guess y_i, for a system of N nodes. We fit coefficients for each set of terms sequentially.

We can place limits on the cost of running this algorithm. To find the best M_i-term description of an output guess, we must find $Q_i(M_i)$ sets of coefficients using Cramer's rule, which can be evaluated in $O(M_i^3)$ time [12]. In the case where there are many more possible terms than we are seeking to use, $M_i \ll NK^2$, this has cost $C_i \approx \frac{(NK^2)^{M_i} M_i^3}{M_i!}$; in

the worst case, where $2M_i = NK^2$, the cost becomes $C_i \approx \left(NK^2\right)^3 2^{NK^2-3}$, though it is difficult to imagine what the utility of such a long description might be to any modellers of very large systems.

The simple L_2 loss function allows us to easily choose the most suitable M_i-term polynomial description of the guess $y_i(t)$. In order to choose the best overall value of M_i, we must develop a new metric: *generalisability*. Our aim is to develop the most accurate possible description of the system using the fewest possible terms; as we increase M_i to infinity, the terms we add will become spurious overfits, increasing the 'accuracy' of the system according to the loss function but failing to actually describe the laws obeyed by the system. We therefore define the generalisability metric: having calculated the best set of coefficients for a given M_i, $\{f_{im}(M_i)\}$, we re-compute them R times and denote these guesses $\{f_{im}(M_i)\}_r$. Each re-computation will involve randomly choosing M_i timepoints; if M_i is large enough to produce an overfit, then the coefficients we generate will be highly dependent on the chosen timepoints, and we will be unable to generate the same values R times. The generalisability for a set of guesses $\{y_i\}$ and polynomial lengths $\{M_i\}$ is defined

$$G\left(\{M_i\}\right) = \sum_{i,m} \frac{\sqrt{\sum_r \left(f_{imr}\left(M_i\right) - \overline{f_{im}}\left(M_i\right)\right)^2}}{\left|\overline{f}_{im}\left(M_i\right)\right|} \tag{26}$$

i.e. the ratio of the standard deviation of recomputations of a weight to the magnitude of its average, summed over all coefficients in the system.
